# Presence of a stridulatory apparatus in the manca stages of isopods (Crustacea, Isopoda, Oniscidea)

**DOI:** 10.3897/zookeys.801.23018

**Published:** 2018-12-03

**Authors:** Giuseppe Montesanto

**Affiliations:** 1 Dipartimento di Biologia, Università degli Studi di Pisa, Pisa 56126, Italy Università degli Studi di Pisa Pisa Italy

**Keywords:** *
Armadillo officinalis
*, biotremology, crustaceans, manca stages, terrestrial isopods

## Abstract

*Armadilloofficinalis* Duméril, 1816 (Armadillidae) is a widespread terrestrial isopod species in the Mediterranean basin and on the western coasts of the Black Sea. The species is adapted to live in xeric environments and has mainly nocturnal habits. This species is capable of producing stridulations, which is nowadays recognized as a synapomorphy of the genus. In both sexes, these vibrations are produced by a line of scales on the propodus of pereopod 4 and 5. The main goals of this study are: to describe the manca stages of *Armadilloofficinalis*; to detect the presence of the stridulatory apparatus in the manca stages; to evaluate the differences of such apparatus in the various manca stages. The manca stages (I, II, III) of *Armadilloofficinalis* are described for the first time showing: i, the shortest duration (known in literature) of the manca stage I (approximately 30 minutes); ii, the presence of a rudimental stridulatory organ that may be of great importance in terms of evolutionary aspects and adaptation to terrestrial life. Notes on the reproductive biology are also reported. Furthermore, some considerations on future perspectives for *A.officinalis* as a model species in biotremology are also discussed.

## Introduction

*Armadilloofficinalis* Duméril, 1816 is a species of terrestrial isopod (Crustacea, Isopoda, Oniscidea) belonging to the family Armadillidae. The genus *Armadillo* Latreille, 1802 is restricted to the Mediterranean basin and western Asia (Schmalfuss 1996, 2003). As defined by Schmalfuss (1996), the species of this genus bear, in both sexes, a line of scales on the propodus of the fourth and fifth pereopod, used for stridulation. This stridulatory apparatus was first observed by Verhoeff (1908) on specimens of *A.officinalis*, but only 70 years later it was described by Caruso and Costa (1976). In addition to this line of scales, the *plectrum*, there are also areas, the so-called *pars stridens*, located on the internal surface of the pereon epimera, consisting of several slightly rounded ridges (approx. 70–80 um long) (Caruso and Costa 1976). The animal produces the sound when it is rolled up, rubbing the *plectrum* against the *pars stridens*. The structures described above have only been recorded in the Mediterranean species of *Armadillosensu stricto* and certainly represent a synapomorphy of this genus (Schmalfuss 1996). The presence of a similar stridulatory organ was also reported by Taiti et al. (1998) in *Cubariseveresti* Vandel, 1973 (Armadillidae) from Nepal, and in two new still undescribed species in the same genus (S. Taiti pers. comm.). The above-mentioned studies do not report whether the stridulatory apparatus appears in the first stages of the post-embryonic development, or later in the juveniles, or if the character is present only in the adults.

In the last years, *A.officinalis* has been the object of several studies in different fields of ethology, ecology, and reproductive biology (Nair et al. 1989, Warburg and Cohen 1992, AlJetlawi and Nair 1994, Warburg 2012, 2013). This species occurs in xeric environments, with mainly nocturnal habits (Vandel 1962), and reproduces several times during its life (iteroparous species). Its reproductive period varies in different geographic areas: in France from June to August (Vandel 1962), in Sicily from May to July (Messina et al. 2011, 2012), and in Israel in October (Warburg 2013). *Armadilloofficinalis* usually lives on several kinds of substrates such as sand, silty-clay substrates, and rocks, as well as in environments populated by different plant communities (Messina et al. 2014).

The feeding preferences and the duration of the stages and substages of the moult cycle of this species was recently studied in detail by Montesanto and Cividini (2017, 2018). Furthermore, a possible use of this species as bioindicator for the exposure to benzene was also examined (Agodi et al. 2015).

Although many of its biological features are well known, the post marsupial manca stages have never been described. The aims of this study are: to describe the manca stages of *Armadilloofficinalis*; to detect the presence of the stridulatory apparatus in the manca stages and to describe it in these different stages.

## Materials and methods

With the aid of forceps, numerous specimens of *Armadilloofficinalis* were collected, under the stones of Catania University campus, eastern Sicily (DMS: 37°31'39"N 15°04'20"E); they were then bred in Pisa (western Tuscany), in a climate room at 20°C, with a natural photoperiod. Ovigerous females were separated from the main livestock and bred separately. Each ovigerous female was kept in a periodically moistened Petri dish (Falcon® 351029, 100×15mm, with plaster of Paris substrate), and fed with slices of potatoes and plane-tree leaves (as in Montesanto and Cividini 2017).

Once the mancas were released from the marsupium they were counted, separated from the female, and raised individually in Petri dishes. The ovigerous females were daily monitored, in order to record the time of manca release. In the same way the postmarsupial mancas were observed every day, so that the moulting process and the time of each manca stage could be monitored and recorded. Twenty-five ovigerous females were dissected in order to study the first manca stage in its intramarsupial development.

Throughout postmarsupial development, twenty individuals, representing each postmarsupial manca stage, were fixed in 70% ethanol for a later analysis. The manca stages were described in accordance with the previously described procedures (Araujo et al. 2004, Brum and Araujo 2007, Sokolowicz et al. 2008, Montesanto et al. 2012), including the appendages morphological study. The parts were mounted on slides, and then pencil drawings were made using a Carl Zeiss Standard 14 microscope equipped with a *camera lucida* (drawing tube). Final illustrations were prepared using the software GIMP ver. 2.8.14 as in Montesanto (2015, 2016). For the SEM (scanning electron microscope), some samples were dried out at room temperature and then covered with a golden film (Edwards Sputter Coater S150B). They were then observed using a Jeol JSM-5410 with a tension of 15 kV; digital images were taken with the Jeol SemAfore system.

## Results

**Figure 1.**
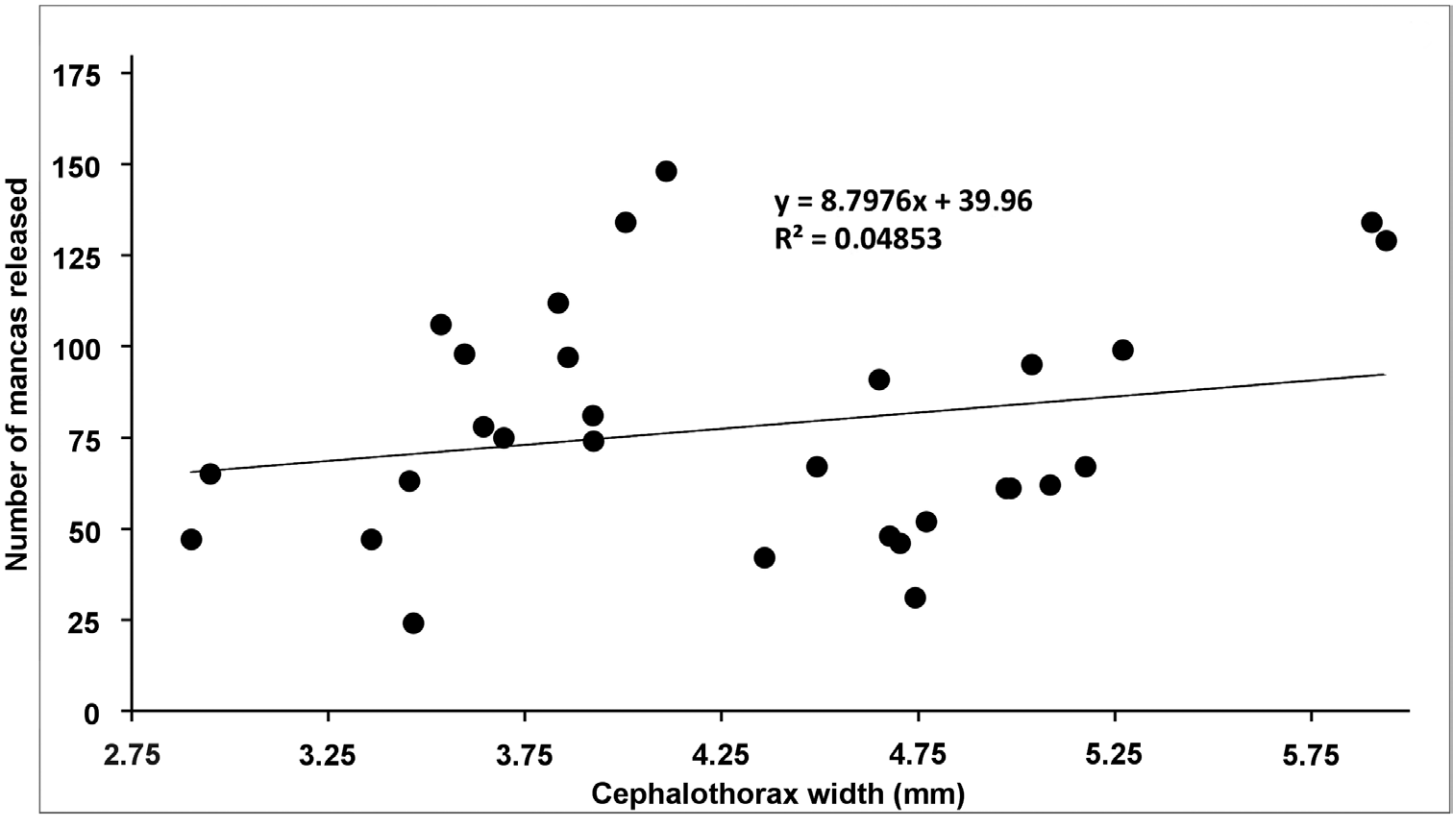
Correlation between cephalothorax widths of the ovigerous females with the number of mancas released.

In order to evaluate the number of mancas released from the marsupium, thirty-two ovigerous females of *Armadilloofficinalis* were considered. Fig. 1 reports the number of mancas released in relation to the female size (cephalothorax width); the values ranged between 25 and 150 mancas.

**Figure 2.**
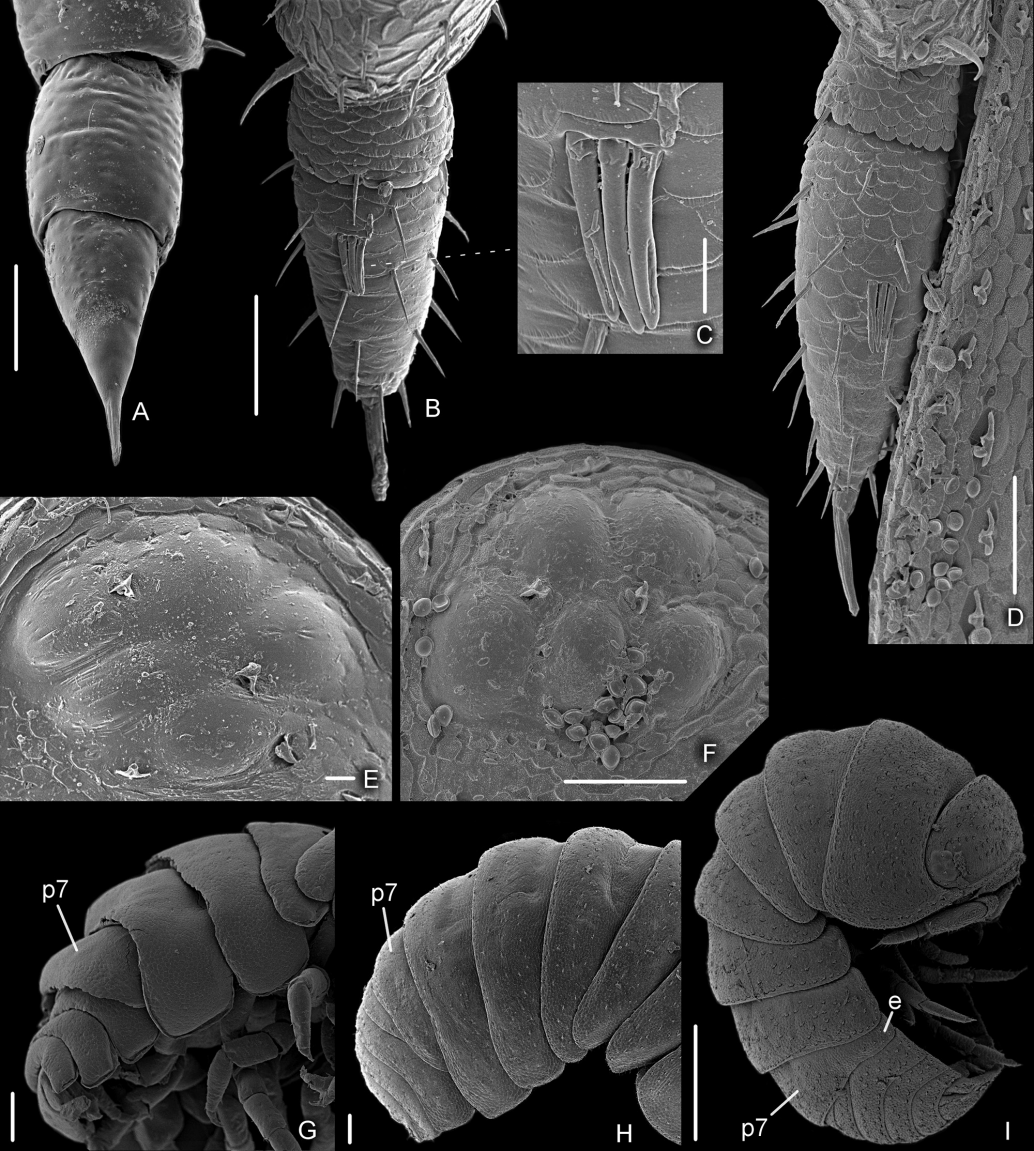
*Armadilloofficinalis* Duméril, 1816. **A** manca stage M I, antennal flagellum **B** manca stage M II, antennal flagellum **C** manca stage M II, aesthetascs on the second article of antennal flagellum **D** manca stage M III, antennal flagellum **E** manca stage M II, eye **F** manca stage M III, eye **G** manca stage M I, p7: pereonite 7 **H** manca stage M II, p7: pereonite 7 **I** manca stage M III, p7: pereonite 7, e: epimeron. Scale bars: 10 μm (**C**, **E**); 50 μm (**A**, **B**, **D**, **F**); 100 μm (**G**, **H**); 500 μm (**I**).

*Armadilloofficinalis* showed three postmarsupial stages (called manca stages M I, M II, and M III), separated by ecdysis and generally characterized by the absence of the first pleopods and non-fuctional seventh pereopods. The following three main characteristics can be used to distinguish each stage: the length of the antennal flagellum articles, the number of ommatidia, the developmental level of the pereopod 7, the presence of the epimera of pereonite 7 (Fig. 2).

**Figure 3.**
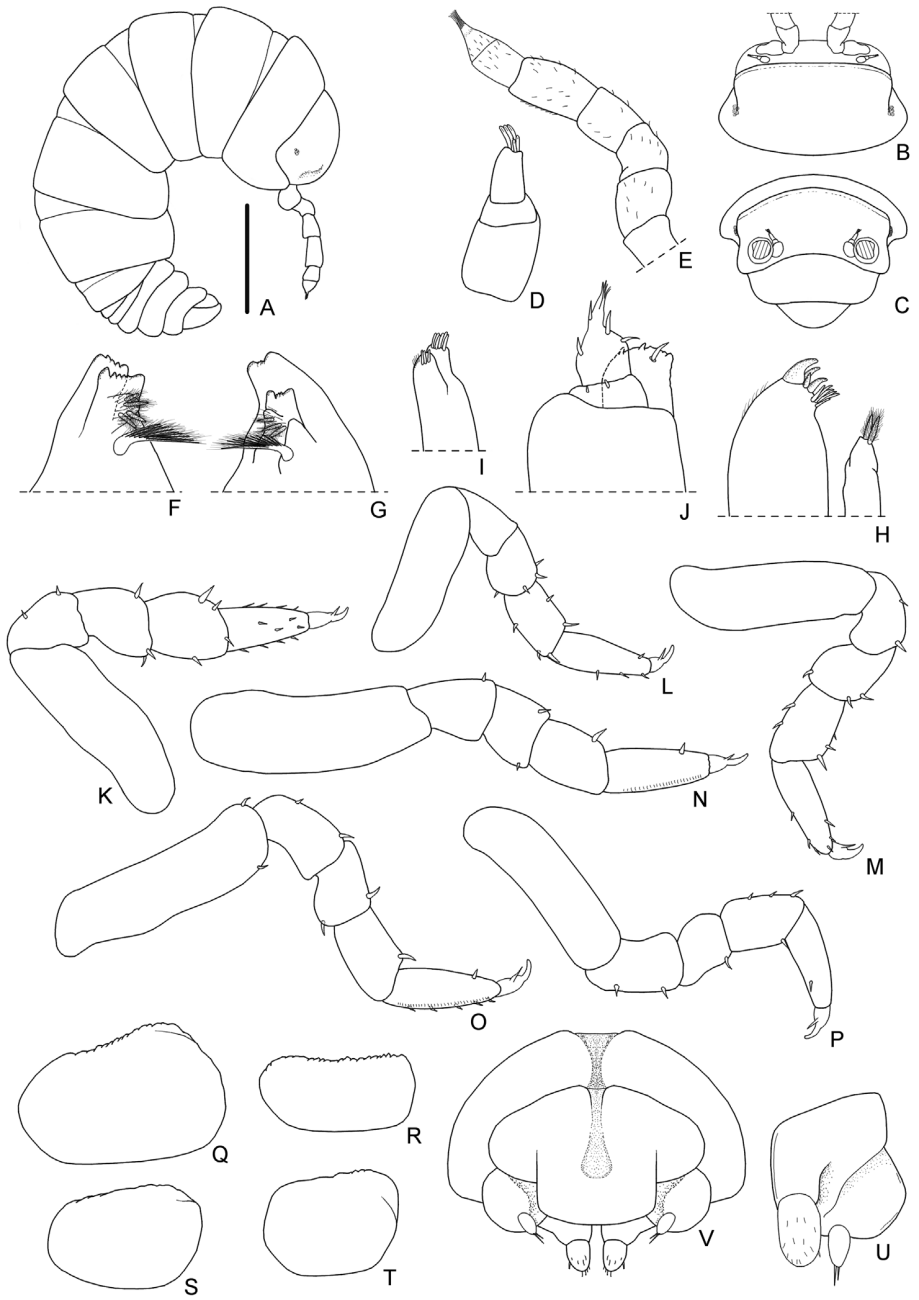
*Armadilloofficinalis* Duméril, 1816. Manca stage M I. **A** body, lateral view (scale bar 0.5 mm) **B** cephalothorax, dorsal view **C** cephalothorax, frontal view **D** antennula **E** antenna **F** left mandible **G** right mandible **H** maxillula **I** maxilla **J** maxilliped **K** pereopod 1 **L** pereopod 2 **M** pereopod 3 **N** pereopod 4 **O** pereopod 5 **P** pereopod 6 **Q** pleopod 2 exopod **R** pleopod 3 exopod **S** pleopod 4 exopod **T** pleopod 5 exopod **U** left uropod **V** telson.

*Manca stage M I.* The duration of this stage varied from a minimum of 21 to a maximum of 43 min, with a mean value of 28 min. The mean body length was 1.76 mm (SD: ± 0.10), with a range from 1.69 to 1.89 mm. The mean cephalothorax width was 0.40 mm (SD: ± 0.07), with a minimum of 0.39 mm and a maximum of 0.46 mm. All the mancas of the first larval stage emerge from the marsupium during the anterior ecdysis process (number of females observed: N = 16) or during the posterior ecdysis (N = 4). No females were observed while giving birth of mancas M I during premoult or intermoult stages. During the ecdysis the mancas remain under their mother’s body and quickly eat both the posterior and anterior exuviae. These mancas had no pigmentation, except for the ommatidia and little brown spots on the pereonite margins; the calcification of the cuticle seems to be incomplete. Because of their body transparency it was possible to observe the exuviae inside the gut. Dorsal surface without scale-setae (see also Fig. 2G). Eyes with 4-5 small pigmented spots, visible under the cuticle and with no external typical structures of ommatidia (Fig. 3A–C). Pereonite 7 incomplete and with no epimera (Figs 2G, 3A). Cephalothorax (Fig. 3B, C) with incomplete frontal line. Antennula (Fig. 3D) of three articles and five apical aesthetascs. Antennal flagellum (Figs 2A, 3E) as long as the fifth article of peduncle, bi-articulated and with the proximal article slightly longer than the apical; no aesthetascs on the flagellum. Mandibles (Fig. 3F, G) with molar penicil semidichotomized, 2+2 free penicils on the left and 1+2 on the right mandible. Maxillula (Fig. 3H) with 4+6 teeth, endite with two long penicils. Maxilla (Fig. 3I) with few setae on the apical part of the lateral and medial lobes. Maxilliped (Fig. 3J) palp with apical setal tuft, and two setae in the basal article; endite with three teeth and one apical seta. Pereopods 1–6 (Fig. 3K–P) with few setae; pereopods 4 and 5 propodus with a line of scales. Pereopods 7 and pleopods 1 absent. Pleopod 2–5 exopods (Fig. 3Q–T) without setae on the margins. Uropods and telson as in Fig. 3U, V.

**Figure 4.**
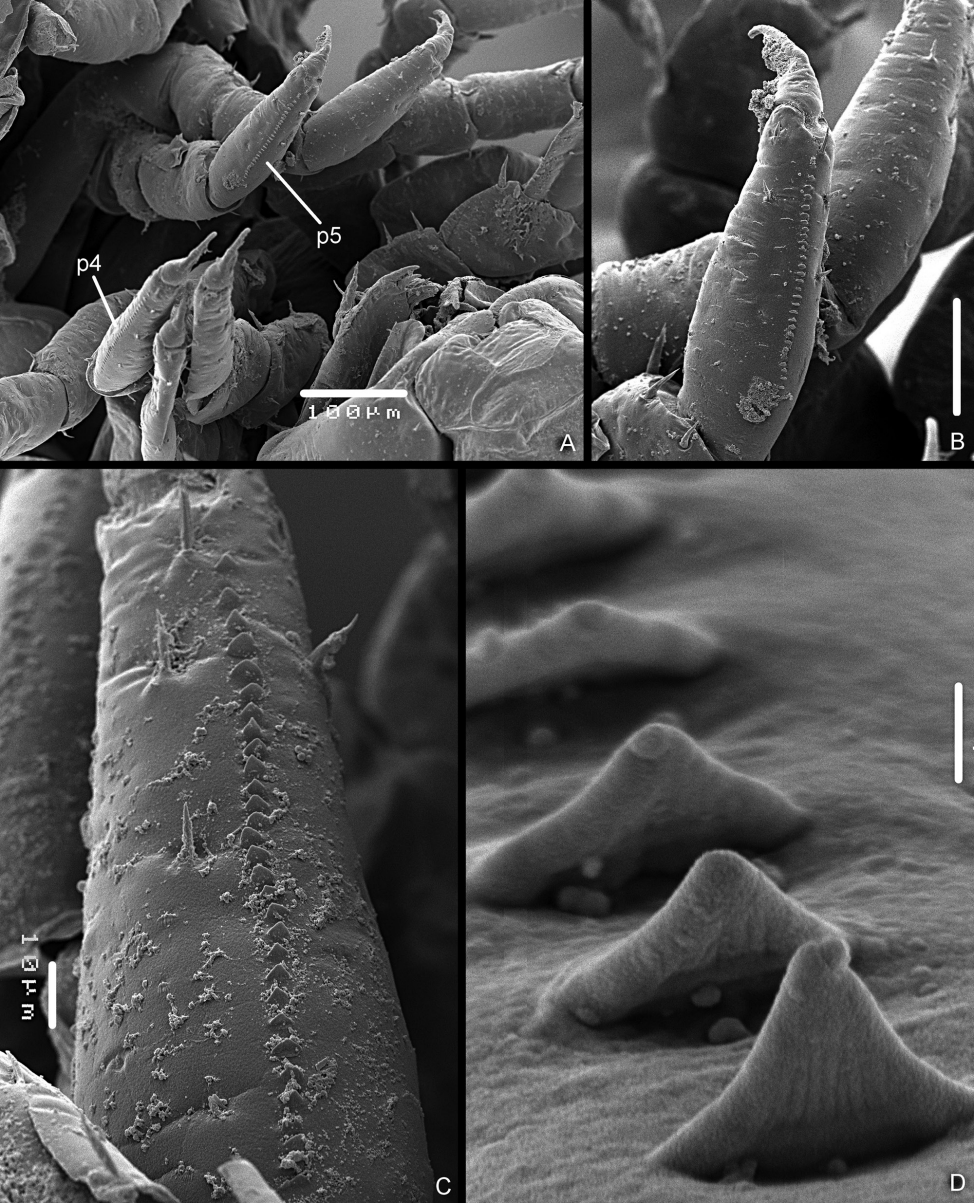
*Armadilloofficinalis* Duméril, 1816. Manca stage M I. **A** pereopod 4 (p4) and pereopod 5 (p5) showing the line of scales on the propodus **B** pereopod 5 propodus, sternal view **C** the line of scales on the pereopod 4 propodus **D** scales of the plectrum on the pereopod 5 propodus. Scale bars: 1 μm (**D**); 10 μm (**C**); 50 μm (**B**); 100 μm (**A**).

*Stridulatory apparatus of Manca stage M I.* Presence of a line of 28–30 scales (*plectrum*) of approx. 100 μm on sternal margin of pereopod 4 and 5 propodus (Fig. 4A–C). Scales of triangular shape (Fig. 4D). No particular modifications of the ventral side of the pereonite 4–6 epimera, no evidence of a specific *pars stridens*.

**Figure 5.**
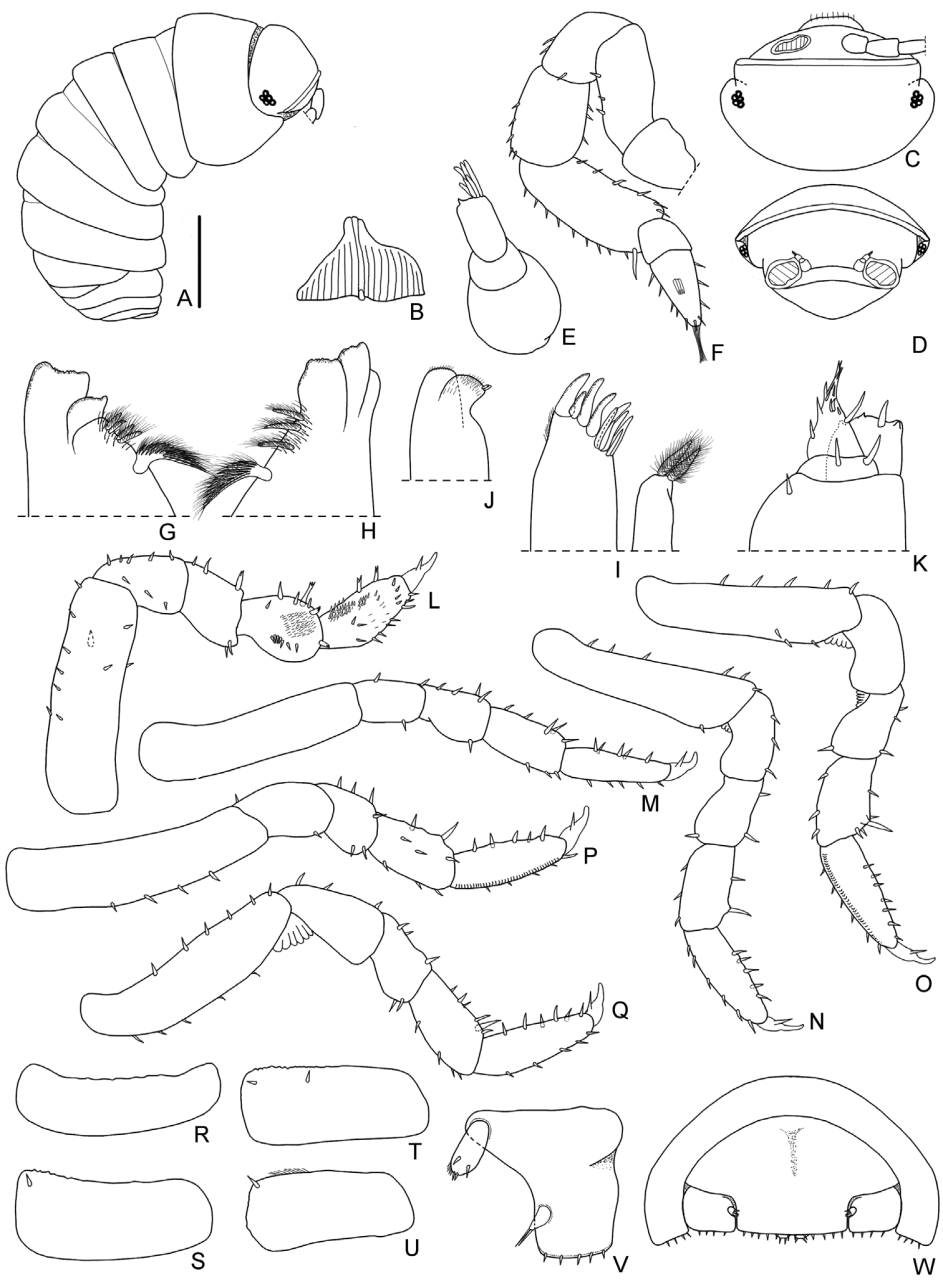
*Armadilloofficinalis* Duméril, 1816. Manca stage M II. **A** body, lateral view (scale bar 0.5 mm) **B** dorsal scale-seta **C** cephalothorax, dorsal view **D** cephalothorax, frontal view **E** antennula **F** antenna **G** left mandible **H** right mandible **I** maxillula **J** maxilla **K** maxilliped **L** pereopod 1 **M** pereopod 2 **N** pereopod 3 **O** pereopod 4 **P** pereopod 5 **Q** pereopod 6 **R** pleopod 2 exopod **S** pleopod 3 exopod **T** pleopod 4 exopod **U** pleopod 5 exopod **V** left uropod **W** telson.

*Manca stage M II.* The duration of this stage varied from a minimum of six to a maximum of seven days, with a mean value of 6.5 days. The mean body length was 2.01 mm (SD: ± 0.04), with a range from 1.92 to 2.11 mm. The mean cephalothorax width was 0.43 mm (SD: ± 0.06), with a minimum of 0.42 mm and a maximum of 0.45 mm. These larvae showed pigmentation on the cephalothorax and the posterior margins of pereonites and pleonites; the calcification of the cuticle seemed to be complete after the previous ecdysis. Even if the body was not completely transparent, a food presence could be observed in the gut as a dark area. At this stage, the mancas left the mother in search of food. Presence of dorsal scale-setae on the body surface (see also Figs 2H, 5B). Eyes with external typical structures of 4–5 ommatidia (Figs 2E, 5A– D). Pereonite 7 still incomplete and with no epimera (Figs 2H, 5A). Cephalothorax (Fig. 5C, D) with a complete frontal line. Antennula (Fig. 5E) of three articles and six apical aesthetascs. Antennal flagellum (Figs 2B, 5F) slightly shorter than the fifth article of peduncle, bi-articulated and with the proximal article shorter than the apical; three aesthetascs on the second article (Figs 2C, 5F). Mandibles (Fig. 5G, H) with molar penicil semidichotomized, 2+2 free penicils on the left and 1+2 on the right mandible. Maxillula (Fig. 5I) with 4+6 teeth, endite with two long apical penicils. Maxilla (Fig. 5J) with few setae on the apical part of the lateral and medial lobes. Maxilliped (Fig. 5K) palp with apical setal tuft, and two setae in the basal article; the endite had three teeth and one apical seta. Pereopods 1–6 (Fig. 5L–Q) with no particular modification but with more setae, in comparison with the previous stage; pereopods 1 with a hairy area on carpus, pereopods 4 and 5 propodus with a line of scales. Pereopods 7 is present although not completely developed. Pleopods 1 absent. Pleopod 2–5 exopods (Fig. 5R–U) with few setae on margins. Uropod and telson as in Fig. 5V, W.

**Figure 6.**
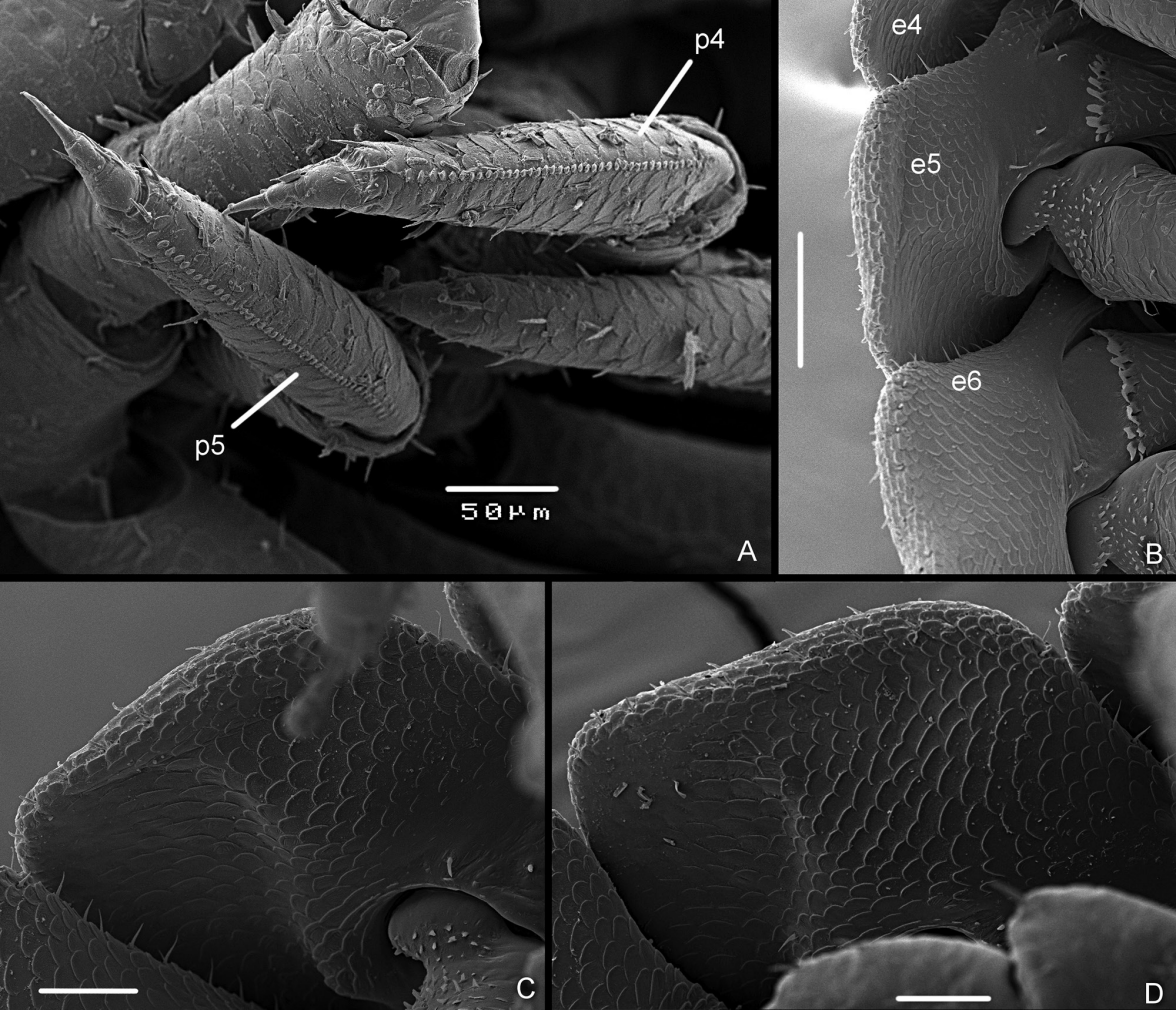
*Armadilloofficinalis* Duméril, 1816. Manca stage M II. **A** pereopod 4 (p4) and pereopod 5 (p5) showing the line of scales on the propodus **B** epimera of pereonite 4 (e4), pereonite 5 (e5), and pereonite 6 (e6), ventral view **C** epimera of pereonite 5, ventral view **D** epimera of pereonite 6, ventral view. Scale bars: 50 μm (**A, C, D**); 100 μm (**B**).

*Stridulatory apparatus of Manca stage M II.* The presence of a line of 38–40 scales (*plectrum*) of approx. 165 μm on sternal margin of pereopod 4 and 5 propodus could be observed (Fig. 6A, C). Scales had a circular shape. No particular modifications of the ventral side of the pereonite 4–6 epimera (Fig. 6B–D), no evidence of the presence of a specific *pars stridens*.

**Figure 7.**
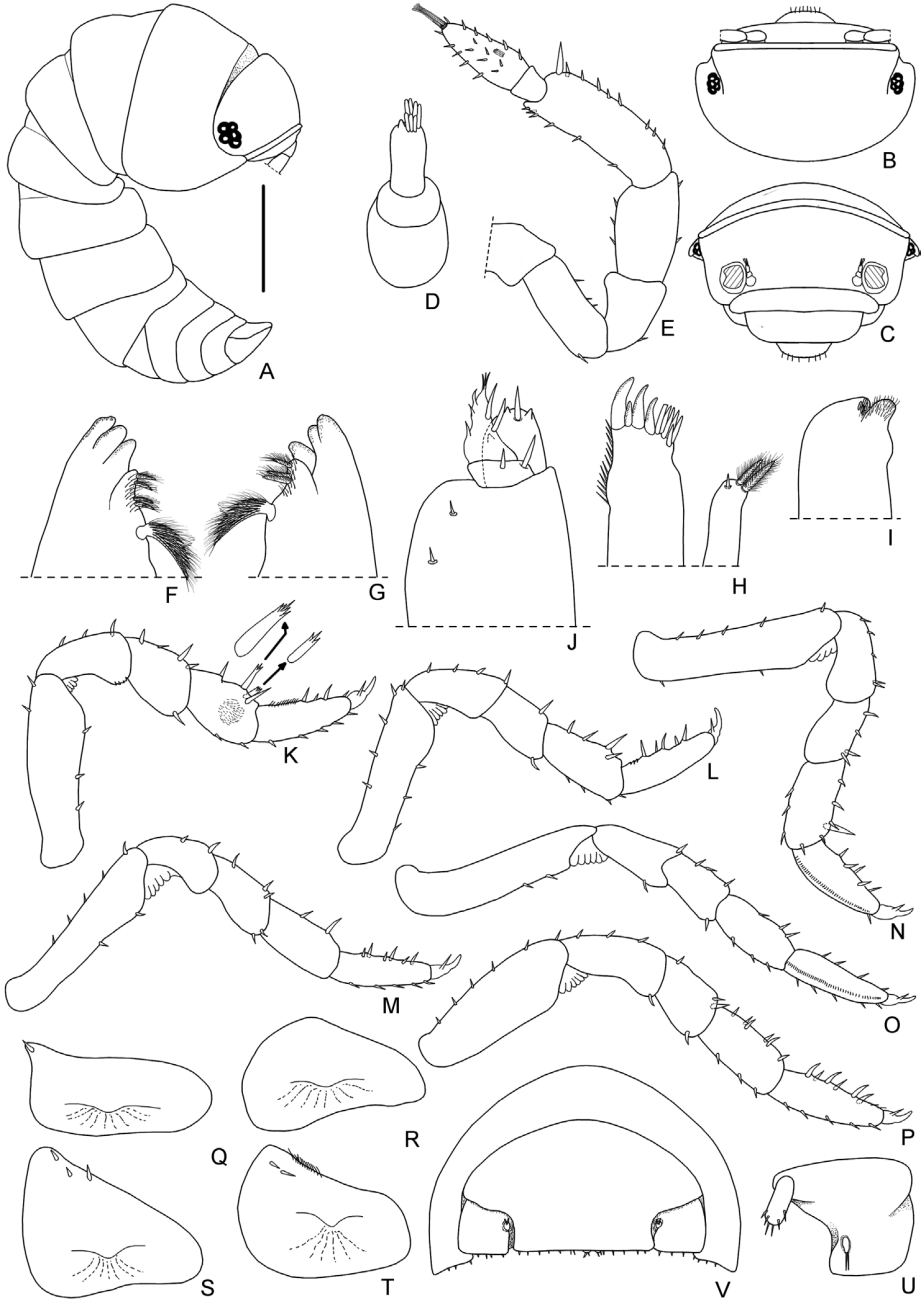
*Armadilloofficinalis* Duméril, 1816. Manca stage M III. **A** body, lateral view (scale bar 0.5 mm) **B** cephalothorax, dorsal view **C** cephalothorax, frontal view **D** antennula **E** antenna **F** left mandible **G** right mandible **H** maxillula **I** maxilla **J** maxilliped **K** pereopod 1 **L** pereopod 2 **M** pereopod 3 **N** pereopod 4 **O** pereopod 5 **P** pereopod 6 **Q** pleopod 2 exopod **R** pleopod 3 exopod **S** pleopod 4 exopod **T** pleopod 5 exopod **U** left uropod **V** telson.

*Manca stage M III.* The duration of this stage varied from a minimum of 20 to a maximum of 24 days, with a mean value of 22 days. The mean body length was 2.35 mm (SD: ± 0.09), with a range from 2.16 to 2.60 mm. The mean cephalothorax width was 0.56 mm (SD: ± 0.08), with a minimum of 0.51 mm and a maximum of 0.59 mm. These larval stage showed more pigmentation than in the previous stage, on the cephalothorax, pereonites, and pleonites; the calcification of the cuticle is complete after the previous ecdysis. Higher presence of dorsal scale-setae on the body surface (see also Fig. 2I). Eyes with external typical structures of 5 ommatidia (Figs 2F, 7A–C). Pereonite 7 completely developed with the presence of epimera (Figs 2I, 7A). Cephalothorax (Fig. 7B, C) with a complete frontal line. Antennula (Fig. 7D) with 3 articles and 7 apical aesthetascs. Antennal flagellum (Figs 2D, 7E) shorter than fifth article of peduncle, bi-articulated and with the proximal article shorter than the apical; 3 aesthetascs on the second article (Figs 2D, 7E). Mandibles (Fig. 7F, G) with molar penicil semi-dichotomized, 2+2 free penicils on the left and 1+2 on the right mandible. Maxillula (Fig. 7H) with 4+6 teeth, endite with two long penicils and one apical seta. Maxilla (Fig. 7I) with few setae on the apical part of lateral and medial lobes. Maxilliped (Fig. 7J) palp with apical setal tuft, and two setae in the basal article; endite with three teeth and one apical seta. Pereopods 1–6 (Fig. 7K–P) with no particular modification but with more setae, if compared with the previous stage; pereopods 1 with a hairy area and tricorn setae on carpus, pereopods 4 and 5 propodus with a line of scales. Pereopods 7 completely developed, showing articles but folded on the pereonal sternites. Pleopods 1 not fully developed. Pleopod 2–5 exopods (Fig. 7Q–T) with few setae on the margins, tracheal fields present but not completely developed. Uropod and telson as in Fig. 7U, V.

**Figure 8.**
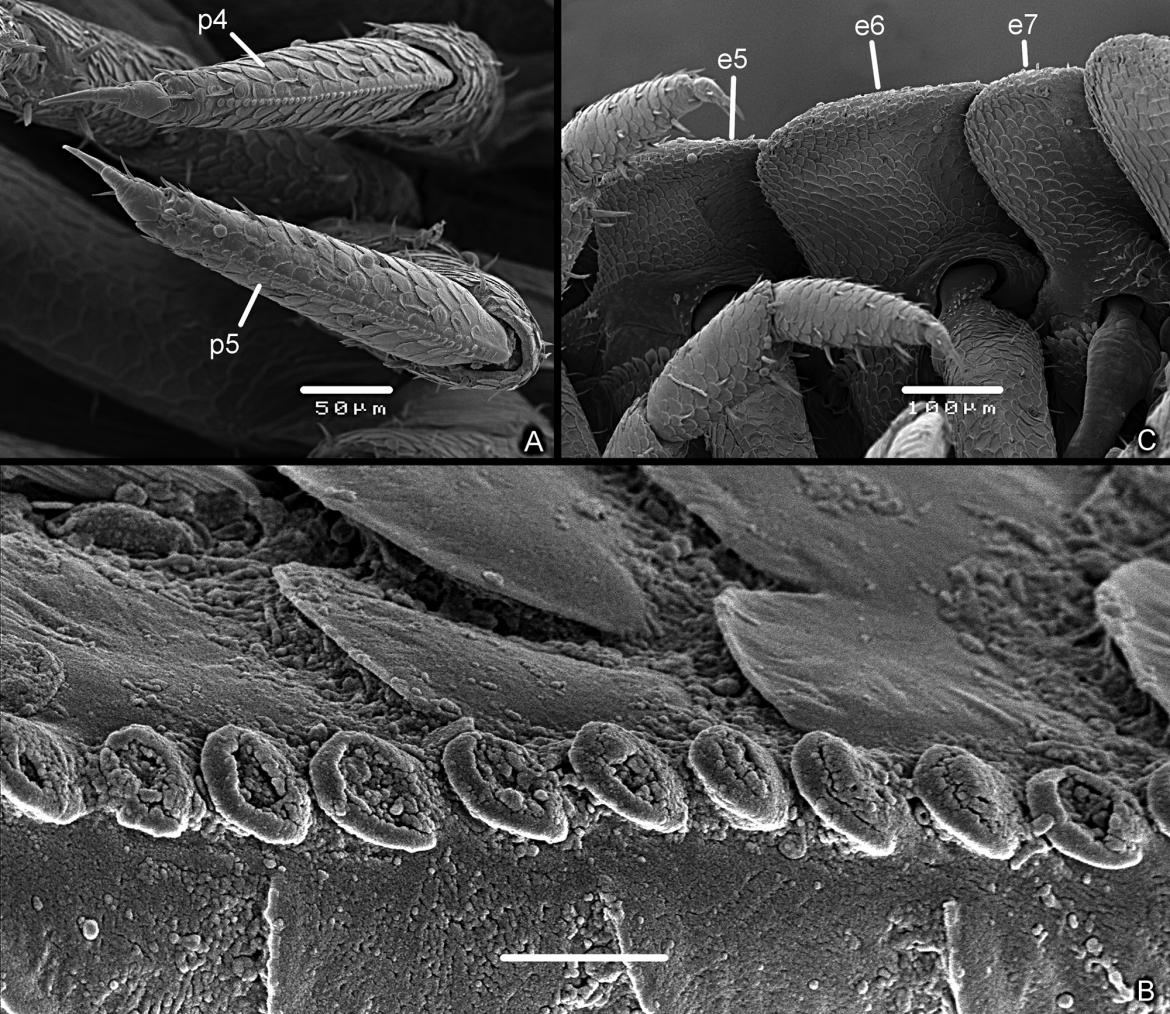
*Armadilloofficinalis* Duméril, 1816. Manca stage M II. **A** pereopod 4 (p4) and pereopod 5 (p5) showing the line of scales on the propodus **B** scales of plectrum of pereopod 4 propodus **C** epimera of pereonite 5 (e5), pereonite 6 (e6), and pereonite 7 (e7), ventral view. Scale bars: 5 μm (**B**); 50 μm (**A**); 100 μm (**C**).

*Stridulatory apparatus of Manca III stage.* Also at this stage it was possible to observe the presence of a line of approx. 40 scales (*plectrum*) of 160 μm on sternal margin of pereopod 4 and 5 propodus (Fig. 8A, B). Scales with a circular shape (Fig. 8B). No particular modifications of the ventral side of the pereonite 4–6 epimera (Figs 8C), no evidence of a specific *pars stridens*.

## Discussion

This detailed study of the morphology of the manca stages of *Armadilloofficinalis* has highlighted their differences. The cephalothorax develops a complete frontal line, passing from M I to manca stage M II. In the antennula the number of apical aesthetascs varies from five on M I to seven on M III. Antennal flagellum changes the proportion of the two articles from M I to M II; the aesthetascs appear on the flagellum in M II and remain the same in M III. Buccal appendages do not modify their main structure in the three manca stages. The number of setae in pereopods increased from M I to M III; pereopod 1 shows a well-defined hairy area on carpus from manca stage M II, pereopods 4 and 5 propodus show a line of scales in the three manca stages, but the number of the scales and their distance on the sternal margin of propodus varies distinctly; pereopods 7 are absent in the first manca stage, there are hints in the second stage, and they are fully developed but ventrally folded in the third manca stage. Pleopods 1 are absent in manca stages M I and M II, but appear in the third manca stage as a hint. Setae on pleopod 2–5 exopods appear in the manca stages M II and they do not considerably increase their number in the next stage. In the three manca stages, the uropod and telson change substantially in shape and proportion.

As for the comparison with other larval stages of terrestrial isopods, the mancas of *A.officinalis* show some significant differences. First of all, as reported for *Atlantosciafloridana* (Van Name, 1940) (Araujo et al. 2004), *Benthanacairensis* Sokolowicz, Araujo & Boelter, 2008 (Sokolowicz and Araujo 2008), and *Porcelliosiculoccidentalis* Viglianisi, Lombardo & Caruso, 1992 (Montesanto et al. 2012) the manca stage M I represents the last marsupial stage, when the mancas do not eat or moult. In the case here showed, the first manca stage begins its moulting process inside the marsupium. The duration of the manca stage is: 48 hours (Kacem-Lachkar 1997) in *Hemilepistusreaumurii* (Milne Edwards, 1840); 19 hours (Brum and Araujo 2007) in *Porcelliodilatatus* Brandt, 1831; 12 hours (Araujo et al. 2004) in *A.floridana*; approx. 6 hours (Zecchini and Montesanto in press), in *Armadillidiumgranulatum* Brandt, 1833; just one hour (Montesanto et al. 2012), in *P.siculoccidentalis*. This duration is even shorter in *A.officinalis*, just half of an hour outside the marsupium, which is the shortest duration among the postmarsupial mancas previously described. This could be considered as an adaptation to the xeric environment, where this species normally lives in the Mediterranean area.

The main body development follows the same rules of the already known oniscidean manca stages: the proportion inversion of the antennal flagellum articles, the development of the seventh pereonite, the appearance of pereopod 7 and pleopod 1. Other minor differences are in the number of ommatidia, and in the number of setae on the margins of pereopods and pleopods (Araujo et al. 2004, Brum and Araujo 2007, Milatovič et al. 2010, Montesanto et al. 2012). The number of mancas released resulted similar to other data reported for *A.officinalis* from Libya (AlJetlawi and Nair 1994), as well as for some neotropical species, such as *A.floridana* (Araujo et al. 2004), *B.cairensis* (Sokolowicz et al. 2008), *Balloniscusglaber* (Araujo & Zardo, 1996), and *Balloniscussellowii* (Brandt, 1833) (Quadros et al. 2008).

The present study has also shown the presence of the stridulatory apparatus, even in *A.officinalis* early stages of development, which is an absolutely new issue for the taxon Oniscidea. The presence of similar apparatus in different taxa of Arthropoda (especially in Insecta) was widely known, even in larval or juvenile stages (Johnstone 1964). The structures such as those on the pereopods of *A.officinalis* are barely known, to the best of my knowledge, in the taxon Crustacea. The *plectrum* morphology could be well described in the larval stages of *A.officinalis*, but the presence of the *pars stridens* (*sensu*Caruso and Costa 1976) was not detected in the three larval stages. This may indicate that the stridulatory apparatus is not yet functional at these developmental stages; however, during the past four years I have never heard in the studied mancae the typical vibrational sound of the adults.

The presence of a such stridulatory apparatus definitely is a synapomorphic character of the genus *Armadillo*. The identification of the typical stridulatory structure of adults in the mancas, even in an early stage development, represents a relevant discovery. Its presence must be, without doubt, the result of a long evolutionary process. It leads to believe that this character, defining the genus *Armadillo* (*sensu*Schmalfuss 1996), might also be present in other genera (Taiti et al. 1998).

It still remains an open question the purpose of a stridulatory apparatus in larval stages, even in biological circumstances in which it could not be used (e.g. inside the maternal brood pouch). I have observed that adults of *A.officinalis* produce stridulation only when their body is rolled up in a ball: this should be a further step in an hypothetic line of a defense strategy or aggregation phenomena. Further studies on these aspects are currently underway. The presence of stridulatory apparatus in larvae of other group of arthropods is well known. Grandi (1951) reported some cases of sound production in preimaginal stages of insects. For stridulation, beside the case of passalid larvae, it is noteworthy the behaviour of other beetle larvae exhibiting stridulatory organs on legs: geotrupid, lucanid, and hybosorid larvae (Palestrini et al. 1990) for instance. Among insects that produce sounds by beating or scrubbing an area of their body against surrounding surfaces, Grandi (1951) reported larvae of the lepidopteran genus *Chimabacche* Zeller, 1839 (gelechiid moths) and pupae of the lepidopteran genus *Eligma* Hübner, 1819 (noctuid moths). The stridulation is also common in *Hydropsyche* Pictet, 1834 larvae (Trichoptera) (see also Johnstone 1964). It could be hypothesized that acustic recalls are audible by the co-specific larvae. So, it is possible that acoustic recalls of larvae that reached a favourable environment may be useful to stimulate other larvae to reach the same environment (Jansson and Vuoristo 1979).

Moreover, other fields of research investigate the possibility of *A.officinalis* to produce substrate-borne vibrations instead of air-borne sounds, as defined in Hill (2009). Recently, some novel aspects have been published, proving the existence of a significant association between alternating turn behaviour and substrate-borne vibrations *in A.officinalis* (Cividini and Montesanto 2018a, 2018b). It has been found that different species of terrestrial isopods, *A.officinalis* and *Armadillidiumvulgare* (Latreille, 1804), show a different reactivity to substrate-borne vibrations, responding with a lower or higher number of turn alternations. This feature might also be involved in the use of substrate-borne vibrations as a means of communication, as in insects. For a better understanding of these so complex phenomena, further research is needed, and *A.officinalis* represents an excellent experimental model to investigate the behaviour of terrestrial isopods in a newly-named discipline known as biotremology (Hill and Wessel 2016, Cividini and Montesanto 2018c).

## Conclusions

Science already knows approximately 3,800 species of terrestrial isopod (Schmalfuss 2003), but larval stages were described only in very few species. Due to many difficulties and technical problems (e.g., related with small larval body size), the study of crustacean larval biology is limited, nevertheless, these fields of research have greatly contributed to improve the knowledge of the life-history of different taxa (ontogeny of species-specific traits), and may have an important and wide appeal in the reconstruction of the phylogeny of higher taxa (“Evo-Devo” perspective) (Anger 2006). A remarkable character that needs to be further studied is the duration of the three postmarsupial manca stages in terrestrial isopods. It highly ranges among the species, especially in the first manca stage, and this should be kept in high consideration in future studies. More data are surely needed, especially in different families aside from the common families Porcellionidae and Armadillidiidae.

With regard to the findings in the present study, the presence of a stridulatory apparatus in a terrestrial crustacean surely constitute an important issue on the terrestrialisation of this taxon (see also Hornung 2011) and may have future implications on the knowledge of the evolutionary biology of the family Armadillidae and the suborder Oniscidea. In the near future, it would be important to understand at what stage of the subsequent development of juveniles stages the stridulatory apparatus becomes functional. These studies are currently underway.
